# Efficacy of the Roy Adaptation Model with smartphone training in reducing urinary tract infection in pediatric clean intermittent catheterization: a prospective study

**DOI:** 10.1007/s00431-024-05924-6

**Published:** 2025-01-14

**Authors:** Canan Sari, Mukaddes Kalyoncu

**Affiliations:** 1 Department of Health Care Services, Elderly Care, Tonya Vocational School, Trabzon University, Tonya, Trabzon, Turkey; 2 Pediatric Nephrology AB, Faculty of Medicine, Karadeniz Technical University, Trabzon, Turkey

**Keywords:** Caregiver, Child, Clean intermittent catheterization, Roy Adaptation Model, Smartphone, Urinary tract infections

## Abstract

**Purpose:**

Study aimed to compare incidence of urinary tract infection (UTI), type of bacteria grown, development of antibiotic resistance over 2 years in children whose caregivers underwent training based on the Roy Adaptation Model with an android phone application for patients clean intermittent catheterization (RAMACIC) versus those whose caregivers received routine training in hospital.

**Method:**

This study was conducted as a descriptive, prospective study with 40 patients and caregivers between October 2021 and 2023 as a continuation of a previously conducted randomized controlled experimental study by the researchers. Data were collected the “Participant Form,” and “Urine Test Form” analyzed with the SPSS 22 package. Descriptive data were determined by number (n), percentage (%), mean, and standard deviation. The frequency of UTI between the two groups was determined by the Chi-square test, and the effect size of the data was determined by Cramer’s V value of the Chi-square test.

**Results:**

A significant reduction in UTI incidence among children of RAMACIC-trained caregivers during the first, second, third, and fourth 6-month periods compared with those under routine CIC training was observed (*p* < 0.05). It was determined that *E. coli* was the most frequently grown bacteria in both groups, with a higher rate in the group receiving routine training in the hospital, and antibiotic resistance was higher in the group receiving routine training in the hospital compared with RAMACIC.

**Conclusion:**

RAMACIC, when administered to caregivers, effectively lowers the long-term risk of UTI development in patients undergoing CIC, also effective in reducing the antibiotic resistance that developed in patients.

## Introduction

Clean intermittent catheterization (CIC) involves the removal of urine through the insertion of a catheter into the bladder under hygienic conditions [1]. CIC is the most effective method used by children with spina bifida (SB), a subgroup of neural tube defects, and patients with neurogenic bladder, urethral stenosis, bladder dysfunction, and their caregivers to protect the urinary system [2, 3]. The utilization of CIC has been associated with long-term improvements in patients’ quality of life, enhanced development of self-confidence and body image, a decrease in mortality rates, and protective effects on the organs comprising the urinary system, notably the kidneys [4–6].

Despite these positive effects of CIC, many complications such as recurrent infections, hematuria, urethritis, bladder perforation, and urethral stricture can be seen in children when the CIC application training is not implemented effectively enough by the healthcare personnel for the caregivers who perform this procedure, when the caregivers do not pay attention to the hygiene rules during the application, when the frequency of CIC application determined by the doctor is not taken into consideration, and when the caregivers are not adapted to the CIC application process by the healthcare personnel [5–8]. UTI is the most common complication in patients undergoing CIC, increasing the risk of permanent kidney damage and the need for renal replacement therapy [9–11]. To address this, studies emphasize the importance of education to enhance caregivers’ knowledge and skills, suggesting that training should be conducted by nurses well-trained in international protocols and guidelines [12, 13].

Nurses’ planning of health education for caregivers based on theories and models is accepted as a guide in creating the scientific content of the profession [15, 16]. Roy Adaptation Model (RAM), developed by Sister Callista Roy and put into practice in 1970, and finally restructured conceptually in 1986 to guide nursing practice, is one of the nursing models used to ensure adaptation of patients and caregivers to the new process they are experiencing [16]. The basic concepts in the model are as follows: human, environment, nurse, and health. The concept of human in the model defines the person who will be adapted to the new process experienced, this person can be a patient or a caregiver. All internal and external factors affecting the adaptation process are called environment. The state of adapting to the new process experienced is defined as health; the positive response of the person to the given warning is defined as “adaptation,” and the negative response is defined as “incompatibility.” The main purpose of the nursing interventions implemented in line with the model is determined as developing effective and positive adaptation, and it is emphasized that the people who provide this adaptation are nurses [13, 14].

There is no protocol/guide widely used throughout the country for CIC training of caregivers in Turkey, and model-based training is not provided for caregivers. While training for caregivers in hospitals in the western regions of the country is provided by nurses, routine training in hospitals in the eastern regions is carried out individually by non-nurse healthcare personnel or individuals assigned by the company selling CIC catheters without a specific protocol/guideline, and nurses do not play an active role in the training process of caregivers [14]. In a randomized controlled experimental study conducted by the same researchers in 2021 in the eastern region of Turkey on caregivers who applied CIC to their children, the knowledge/skill, coping adaptation level and the frequency of UTI development in children were evaluated after the training given to the caregivers [14]. In the study, caregivers were randomly divided into experimental and control groups. During the study period, routine training given in the hospital was applied to the caregivers in the control group. This training is usually provided by a person working for the company that sells the CIC catheter and who is not a healthcare professional, or sometimes by a service doctor. The training provided only includes verbal explanations and practical applications regarding CIC in the patient room. Nurses do not play an active role as health personnel in the routine training process provided in the hospital [14]. On the other hand, caregivers in the experimental group were given CIC training supported by an android phone application based on the Roy Adaptation Model, abbreviated as RAMACIC by the researchers. This training, structured by the researchers according to the physiological, self-concept, role-function, and interdependence domains of RAM, was implemented in the patient room for 4 days between 16:00 and 18:00 using a face-to-face training model and a power point presentation [14]. At the end of each training, a urinary catheterization model appropriate for the child’s gender was first used, and then CIC skill practice training was given to the caregiver and the child. After the training was completed, an android phone application designed by the researchers and written by a computer engineer was downloaded to the caregivers’ phones. The purpose of this android phone application designed by researchers is to ensure that the caregiver performs CIC using the correct procedure steps, as frequently as the doctor recommends, and to remind the caregiver of hospital appointments [14]. In the literature review, it was determined that a similar android phone application that included the process steps, CIC application times, and hospital appointments for caregivers who applied CIC to their children had not been used before. This application was created entirely by the researchers and an international patent application was also filed. In order to test the validity and reliability of the designed android application, the application was tested by installing it on the phones of three caregivers who applied CIC to their children but were not included in the study. Software adjustments were made to the application based on feedback from caregivers and then re-installed on their phones. After positive feedback from caregivers, researchers decided to use the android phone application [14]. In addition, since the CIC application process steps differ according to gender, the android phone application has been prepared separately for girls and boys with the same basic program. Within the scope of the study, the patients in the experimental group were revisited by the researchers 1 day before discharge, and the training book was delivered to the caregivers. After discharge from the hospital, a home visit and three telephone interviews were conducted to determine the problems experienced by caregivers regarding CIC. As a result of this study, it was determined that the knowledge/skill, coping/adaptation levels of caregivers who received RAMACIC increased and the incidence of UTI in children within 3 months after the training decreased compared with those who received routine training in the hospital [14].

However, the long-term impact of RAMACIC on caregivers and its influence on UTI development in children remains unknown. The aim of this study was to compare the incidence of UTI, the type of bacteria grown, and the development of antibiotic resistance in children over a 2-year period following RAMACIC given by nurses to caregivers who applied CIC to their children and CIC training routinely applied in the hospital. To our knowledge, this is the first study to evaluate the long-term effects of education given to caregivers who perform CIC on their children using different methods on the development of UTI, bacterial growth, and antibiotic resistance in children with CIC.

The study hypotheses are as follows:

- H_0_: There is no significant difference between the children of caregivers who received RAMACIC given by nurses and the children of caregivers who received routine education in the hospital in terms of the development of UTI, bacterial growth, and antibiotic resistance within 2 years.
- H_1_: There is a significant difference between the children of caregivers who received RAMACIC given by nurses and the children of caregivers who received routine education in the hospital in terms of the development of UTI, bacterial growth, and antibiotic resistance within 2 years.

## Materials and methods

### Study design

**Fig. 1:**
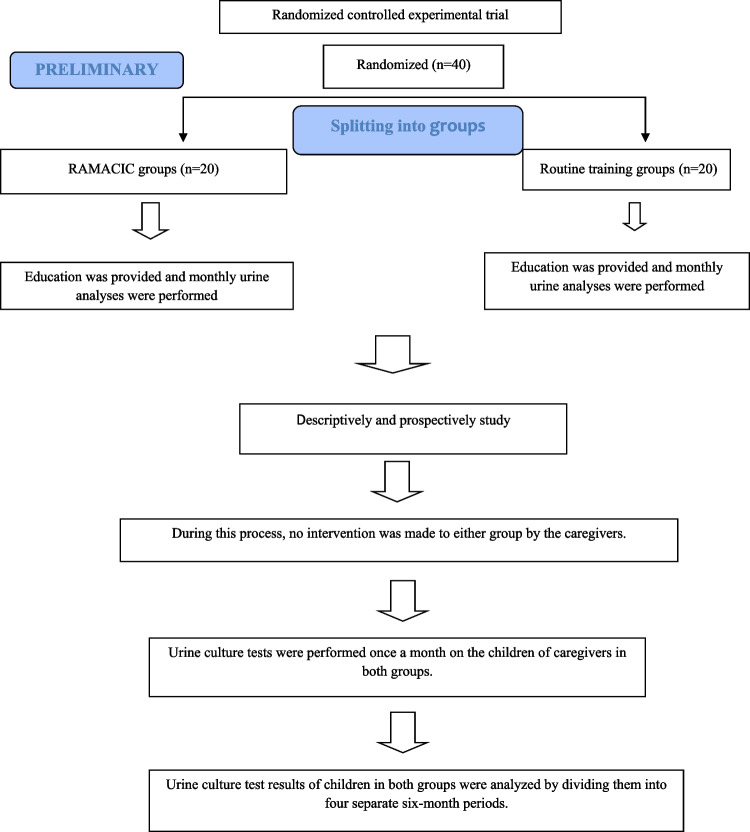
workflow plan of the study

This study aimed to determine the long-term effectiveness of a randomized controlled experimental study evaluating RAMACIC, which was previously conducted and completed by the researchers. No intervention/education was applied by the researchers to either the RAMACIC or the group receiving routine training in the hospital during the 2 years the study was conducted. During this process, only the frequency of UTI development, the type of causative bacteria and antibiotic resistance were evaluated in children in both groups for 2 years. Therefore, the study was conducted descriptively and prospectively. The workflow plan of the study is presented in Fig. 1.

### Population, sample, and sampling method

This study was conducted in the pediatric nephrology department of a university hospital in the eastern part of Turkey between October 1, 2021, and October 30, 2023. The sample of the study consisted of 40 patients (20 experimental, 20 control), who were the sample of a randomized controlled experimental study previously conducted by the researchers [14]. This sample size was calculated according to the variance analysis effect size determined using the G*Power 3.1 program within the scope of the randomized controlled experimental study previously conducted by the researchers [17, 18].

### Data collection

**Fig. 2:**
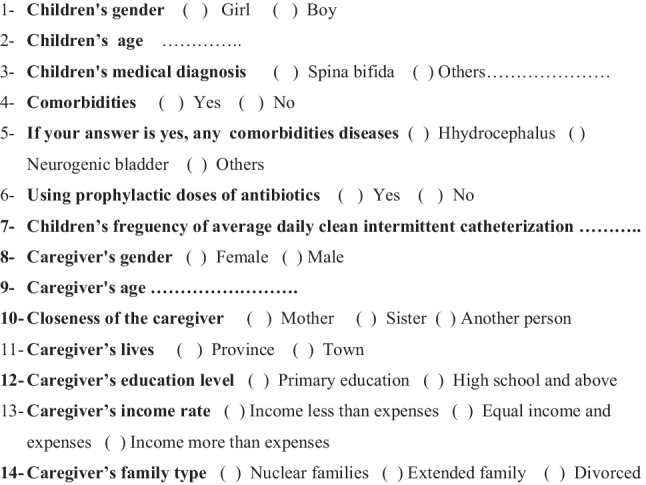
Sociodemographic characteristics of children and caregivers (participant form)

**Fig. 3:**
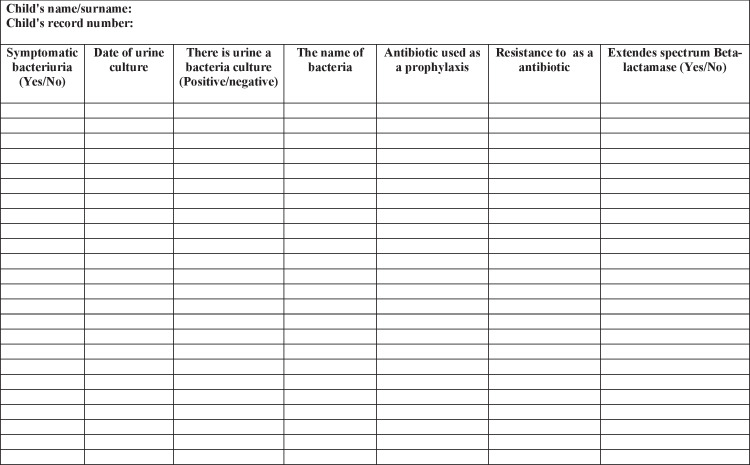
Sociodemographic characteristics of children and caregivers (urine test form)

Study data were collected using the “Participant Form” and “Urine Test Form.” Both forms are presented in Fig. 2 and Fig. 3.

### Participant form

Developed by the researchers in line with the literature, this questionnaire covered sociodemographic characteristics, medical diagnosis, comorbidities, and the frequency of CIC [5–14].

### Urine test form

The patients’ urine culture results were obtained from the hospital records. In this form, the presence of symptomatic bacteriuria, the date of urine culture, the presence of bacteria in the urine, the name of the bacteria if bacteria were seen, the name of the antibiotic used as prophylaxis, the development of resistance to the antibiotic, and the presence of extended spectrum beta-lactamase (ESBL) were questioned. In the hospital where the study was conducted, it is routine practice to use prophylactic antibiotics in children applying CIC and to perform monthly urine culture tests to detect UTI. Additionally, if the patients admitted to the hospital with complaints of fever, hematuria, abdominal pain, and foul-smelling urine, they evaluated as a symptomatic and urine culture was studied. In case of resistance, the antibiotic used was changed according to the results of antibiogramme. Thus, patients used routine prophylactic antibiotics throughout the study period. Since urine samples were collected using CIC, urine culture showing 10,000 or more colonies per milliliter was considered UTI [19, 20]. The development of resistance to antibiotics used in prophylactic doses by patients was determined by the ESBL value. CIC is applied to all patients included in the study by entering the urethra with a disposable catheter. In order to evaluate ITU, bacterial growth, and resistance development in children, urine cultures were taken from all patients by entering the urethra with a disposable catheter sample.

### Ethical considerations

The study received ethical approval from the Karadeniz Technical University Faculty of Medicine Scientific Research Ethics Committee (Decision Number 24237859–86). Participants were verbally informed about the study, and both verbal and written informed consent were obtained from those willing to participate. The study was conducted in accordance with the Declaration of Helsinki.

### Statistical analysis

Data analysis was performed using the Statistical Package for Social Science (SPSS) 22 program. Descriptive data, including sociodemographic characteristics of participants, frequency of UTI development in children, symptomatic UTI, antibiotic resistance, ESBL development, and the types of bacteria identified were analyzed using numerical values (n), percentages (%), mean, and standard deviation. The comparison of UTI development frequency in children between the two groups was assessed using the chi-square test. Cramer’s V value from the chi-square test was employed as the effect size measure for the descriptive data. The effect size was interpreted based on Cohen’s “r” coefficient classification, where r values of 0.10 indicated a low effect, 0.30 a medium effect, and 0.50 a high effect [17, 18].

## Results

Table 1 presents the sociodemographic characteristics of the children and caregivers who participated in the study. In the RAMACIC group, the mean age of the caregivers was 39.90 ± 5.91 years (27–48), the mean age of the children was 7.40 ± 4.23 years (3–18), and the mean daily CIC frequency was 5.35 ± 1.42 (3–10). In contrast, the mean age of the caregivers in the receiving routine hospital education was 36.80 ± 6.97 years (25–48), the mean age of the children was 9.40 ± 4.54 years (4–18), and the mean daily CIC frequency was 4.90 ± 1.25 (3–8) (Table 1).

**Table 1:** The comparison of the sociodemographic characteristics of children and caregivers receiving RAMACIC and routine hospital education

Variable	Group of receiving RAMACIC		Group of routine hospital education		X^2^; p
	*n*	%	*n*	%	
Children’s gender					
Girl	16	80.0	15	75.0	0.143; 0.500
Boy	4	20.0	5	25.0	
Children’s medical diagnosis					
Spina bifida	12	60.0	14	70.0	6.000; 0.112
Neurogenic bladder	8	40.0	6	30.0	
Comorbidities					
Yes	13	65.0	14	73.6	0.058; 0.810
No	7	35.0	5	26.4	
Hydrocephalus	10	86.6	10	71.4	
Biotidinase enzyme deficiency	0	0	1	7.1	
Others*	3	13.4	3	21.5	
Using prophylactic doses of antibiotics					
Yes	19	95.0	20	100.0	1.026; 0.311
No	1	5.0	0	0.0	
Name of antibiotic used					
Nitrofurantoin	11	57.9	14	70.0	0.147; 0.385
Others**	8	42.1	6	30.0	
Caregiver’s gender					
Female	20	100.0	20	100.0	
Closeness of the caregiver					
Mother	19	95.0	20	100.0	1.026; 0.311
Sister	1	5.0	0	0	
Caregiver’s lives					
Province	4	20.0	5	25.0	0.000; 1.000
Town	16	80.0	15	75.0	
Caregiver’s education level					
Primary education	10	50.0	12	60.0	0.001; 0.751
High school and above	10	50.0	8	40.0	
Caregiver’s income rate					
Income less than expenses	10	50.0	6	30.0	1.688; 0.430
Equal income and expenses	8	40.0	11	55.0	
Income more than expenses	2	10.0	3	15.0	
Caregiver’s family type					
Nuclear families	15	75.0	16	80.0	1.924; 0.382
Extended family	5	25.0	3	15.0	
Divorced	0	0.0	1	5.0	
The frequency of daily CIC for the child					
1–5	11	55.0	14	70.0	1.052; 0.591
6–10	9	45.0	6	30.0	
The child’s diaper usage status					
Yes	19	95.0	18	90.0	0.360; 0.500
No	1	5.0	2	10.0	
Fecal incontinence in a child					
Yes	17	85.0	13	65.0	2.133; 0.137
No	3	15.0	7	35.0	
Constipation in a child					
Yes	11	55.0	12	60.0	0.102; 0.500
No	9	45.0	8	40.0	
Children’s mean age mean ± SD (min.–max.)	7.40 ± 4.23 (3–18)		9.40 ± 4.54 (4–18)		
Caregiver’s mean age mean ± SD (min. –max.)	39.90 ± 5.91 (27–48)		36.80 ± 6.97 (25–48)		
Children’s frequency of average daily CIC	5.35 ± 1.42 (3–10)		4.90 ± 1.25 (3–8)		

RAMACIC: based on the Roy Adaptation Model with an android phone application for patients on clean intermittent catheterization, *CIC* clean intermittent catheterization. Others*: vesicoureteral reflux, megacolon, hydronephrosis. Others**: Sefuroksim, trimetoprim, and sulfametoksazol

RAMACIC groups, 80% of the children were girls, 60% had a medical diagnosis of SB, 95% were using antibiotics, 55% had a daily CIC frequency of 1–5, 95% were using diapers, 85% had fecal incontinence, and 55% had constipation. All of the caregivers in the experimental group were women, 95% were the mothers of the children, 80% lived in the town, 50% had less income than expenses and had primary education, and 75% lived in a nuclear family.

Routine training groups, 75% of the children were girls, 60% had a medical diagnosis of SB, all use antibiotics, 70% had a daily CIC frequency of 1–5, 90% were using diapers, 65% had fecal incontinence, and 60% had constipation. All of the caregivers in the routine training group were women, all of the child’s mother, 75% lived in the town, 30% had less income than expenses, 60%had primary education, and 80% lived in a nuclear family (Table 1).

In the study, the sociodemographic characteristics of the caregivers in the RAMACIC group and the group receiving routine training in the hospital were compared using the chi-square test for confounding factors such as gender, medical diagnosis, children having additional diseases, antibiotic use, fecal incontinence, and constipation. No significant difference was found between the two groups in the statistical analysis (Table 1).

In Table 2, the incidence of UTI in children over 2 years, categorized according to 6-month periods, the name of the causative bacteria, symptomatic UTI, antibiotic resistance, and ESBL positivity status, is compared in both groups.

**Table 2:** The comparison of the presence of UTI in children, the causative bacterial species, symptomatic UTI, antibiotic resistance, and ESBL positive between groups in a 6-month period within 2 years

		Group of receiving RAMACIC		Group of routine hospital education		*X*^2^	*p*	*r*
		*n*	%	*n*	%			
UTI on first 6 months in child	Yes	8	40.0	15	75.0	4.949	0.026	0.402
	No	12	60.0	5	25.0			
The name of the bacteria	*Escherichia coli*	8	50.0	11	42.3			
	*Pseudomonas aeruginosa*	2	12.5	3	11.6			
	*Klebsiella pneumoniae*	4	25.0	7	26.9			
	*Enterococcus faecium*	2	12.5	4	15.3			
	*Proteus mirabilis*	0	0.0	1	3.9			
Symptomatic UTI	Yes	6	37.5	15	57.6			
	No	10	62.5	11	42.4			
Antibiotic resistance	Yes	3	18.7	12	46.1			
	No	13	81.3	14	53.9			
ESBL positive	Yes	2	12.5	7	26.9			
	No	14	87.5	19	73.1			
UTI on second 6 months in child	Yes	9	45.0	16	80.0	5.227	0.022	0.0361
	No	11	55.0	4	20.0			
The name of the bacteria	*Escherichia coli*	9	52.9	16	47.3			
	*Pseudomonas aeruginosa*	2	11.7	2	5.8			
	*Klebsiella pneumoniae*	3	17.6	14	41.1			
	*Enterococcus faecium*	2	11.7	1	2.9			
	*Proteus mirabilis*	1	6.1	1	2.9			
Symptomatic UTI	Yes	2	11.8	9	26.5			
	No	15	88.2	25	73.5			
Antibiotic resistance	Yes No	6 11	35.3 64.7	10 24	29.5 70.5			
ESBL positive	Yes	5	29.5	12	35.3			
	No	12	70.5	22	64.7			
UTI on third 6 months in child	Yes	6	30.0	14	70.0	8.182	0.004	0.503
	No	14	70.0	6	30.0			
The name of the bacteria	*Escherichia coli*	2	28.6	19	63.3			
	*Pseudomonas aeruginosa*	0	0.0	3	10.0			
	*Klebsiella pneumoniae*	3	42.8	7	23.3			
	*Enterococcus faecium*	0	0.0	1	3.4			
	*Staphylococcus aureus*	2	28.6	0	0.0			
Symptomatic UTI	Yes	0	100.0	25	83.3			
	No	7	0.0	5	16.7			
Antibiotic resistance	Yes No	1 6	14.3 85.7	6 24	20.0 80.0			
ESBL positive	Yes	1	14.3	5	16.7			
	No	6	85.7	25	83.3			
UTI on fourth 6 months in child	Yes	5	25.0	13	65.0	6.465	0.011	0.402
	No	15	75.0	7	45.0			
The name of the bacteria	*Escherichia coli*	5	83.4	9	36.2			
	*Pseudomonas aeruginosa*	0	0.0	6	24.1			
	*Klebsiella pneumoniae*	1	16.6	6	24.1			
	*Proteus mirabilis*	0	0	2	7.8			
	*Enterococcus faecium*	0	0.0	2	7.8			
Symptomatic UTI	Yes	5	83.4	15	60.0			
	No	1	16.6	10	40.0			
Antibiotic resistance	Yes No	1 5	20.0 80.0	5 20	20.0 80.0			
ESBL positive	Yes No	1	20.0	4	16.0			
		5	80.0	21	84.0			

RAMACIC: based on the Roy Adaptation Model with an android phone application for patients on clean intermittent catheterization, *CIC* clean intermittent catheterization, *UTI* urinary tract infection, *ESBL* extended spectrum beta-lactamase

During the first 6-month period, UTI incidence was 40% in children of caregivers receiving RAMACIC, compared with 75% in those receiving routine hospital education. The decrease in UTI incidence among the children of recipients receiving RAMACIC was statistically significant with a moderate effect size (*r*: 0.402, *p* < 0.026) (Table 2).

In the patients in the receiving RAMACIC group, 37.5% had symptomatic bacteriuria, 18.7% had antibiotic resistance, and 12.5% ​​were ESBL positive. The patients in the receiving routine hospital education group, 57.6% had symptomatic bacteriuria, 46.1% had antibiotic resistance, and 26.9% ​​were ESBL positive (Table 2).

At the end of the first 6 months, urine cultures of children of caregivers taking RAMACIC revealed growth of *Escherichia coli* in 50%, *Klebsiella pneumoniae* in 25%, *Pseudomonas aeruginosa*, and *Enterococcus faecium* in 12.5%. In contrast, 42.3% of the children in the routine education group exhibited *Escherichia coli,* 26.9% *Klebsiella pneumoniae,* 11.6% *Pseudomonas aeruginosa*, 15.3% *Enterococcus faecium*, and 3.9% *Proteus mirabilis* (Table 2).

In the second 6-month period, UTI incidence was 45% in the RAMACIC group and 80% in the routine education group. The decrease in UTI incidence among the RAMACIC group was statistically significant with a moderate effect size (*r*: 0.361, *p* < 0.022) (Table 2).

The patients in the receiving RAMACIC group, 11.8% had symptomatic bacteriuria, 35.3% had antibiotic resistance, and 29.5% ​​were ESBL positive. The patients in the receiving routine hospital education group, 26.5% had symptomatic bacteriuria, 29.5% had antibiotic resistance, and 35.3% ​​were ESBL positive. (Table 2).

At the end of the second 6 months, urine cultures of children of caregivers taking RAMACIC revealed growth of *Escherichia coli* in 52.9%, *Klebsiella pneumonia* in *17.6%, Pseudomonas aeruginosa* and *Enterococcus faecium* in 11.7%, and 6.1% *Proteus mirabilis*. In contrast, 47.3% of the children in the routine education group exhibited *Escherichia coli,* 41.1% *Klebsiella pneumoniae,* 5.8% *Pseudomonas aeruginosa*, and 2.9% *Enterococcus faecium* and *Proteus mirabilis* (Table 2).

In the third 6-month period, the incidence of UTI in children of caregivers receiving RAMACIC was 30%, while in those undergoing routine training in the hospital, it was 70%. The decrease in UTI incidence among the RAMACIC group at the end of this period compared with the other group was significant with a moderate effect size (*r*: 0.503, *p* < 0.004) (Table 2).

The patients in the receiving RAMACIC group had no symptomatic bacteriuria; 14.3% had antibiotic resistance and ESBL positive. In the patients in the receiving routine hospital education group, 83.3% had symptomatic bacteriuria, 20% had antibiotic resistance, and 16.7% ​​were ESBL positive (Table 2).

Bacterial analysis revealed that *Escherichia coli 28.6%, Klebsiella pneumonia 42.8%*, and *Staphylococcus aureus* were present in 28.6% of the urine samples from the RAMACIC group. Conversely, children from the routine training group exhibited *Escherichia coli* in 63.3%, *Klebsiella pneumonia in 23.3%*, *Pseudomonas aeruginosa* in 10.0%, and *Enterococcus faecium* in 3.4% of their urine samples (Table 2).

During the fourth 6-month period, the incidence of UTI in children of caregivers receiving RAMACIC was 25%, while in those undergoing routine training in the hospital, it was 65%. The decrease in UTI incidence among the RAMACIC group at the end of this period compared with the other group was significant with a moderate effect size (*r*: 0.402, *p* < 0.011) (Table 2).

In the patients in the receiving RAMACIC group, 84.3% had symptomatic bacteriuria and 20% had antibiotic resistance and ESBL positive. In the patients in the receiving routine hospital education group, 60% had symptomatic bacteriuria, 20% had antibiotic resistance, and 16% ​​were ESBL positive (Table 2).

Bacterial analysis indicated that *Escherichia coli* in 83.4% and *Klebsiella pneumonia* in 16.6% were detected in the RAMACIC group, respectively. On the other hand, 36.2% of the children from the routine training group had *Escherichia coli*, *Klebsiella pneumonia and Pseudomonas aeruginosa in* 24.1%, and *Proteus mirabilis* and *Enterococcus faecium* in 7.8% in their urine samples (Table 2).

Table 3 compares the names of the causative bacteria of UTI, symptomatic UTI, antibiotic resistance, and ESBL positivity in children in both groups over 2 years. *Escherichia coli* was the most frequently isolated species in both groups. The distribution of bacterial growth in children whose caregivers received RAMACIC within 2 years included *Escherichia coli* (52.1%), *Klebsiella pneumonia* (23.9%), *Pseudomonas aeruginosa* (8.6%), *Enterococcus faecium* (8.6%), *Proteus mirabilis* (2.1%), and *Staphylococcus aureus* (4.7%). In contrast, in children whose caregivers received routine hospital training, *Escherichia coli* accounted for 53.3%, *Klebsiella pneumonia* (22.4%), *Pseudomonas aeruginosa* (13.5%), *Enterococcus faecium* (7.7%), and *Proteus mirabilis* (3.1%) (Table 3). Symptomatic UTI was seen in 28.2%, antibiotic resistance was seen in 23.9%, and ESBL positive was seen in 19.5% in the RAMACIC group over 2 years. On the other hand, in the group receiving routine training in the hospital, symptomatic UTI was seen at a rate of 55.6%, antibiotic resistance at a rate of 28.6%, and ESBL positivity at a rate of 25.9% over 2 years (Table 3).

**Table 3:** The comparison of the presence of UTI in children, the causative bacterial species, symptomatic UTI, antibiotic resistance, and ESBL positive between groups in within 2 years

		Group of receiving RAMACIC		Group of routine hospital education	
		*n*	%	*n*	%
The type of bacteria for two years causes UTI	*Escherichia coli*	24	52.1	55	53.3
	*Pseudomonas aeruginosa*	4	8.6	14	13.5
	*Klebsiella pneumoniae*	11	23.9	24	22.4
	*Enterococcus faecium*	4	8.6	8	7.7
	*Proteus mirabilis*	1	2.1	2	3.1
	*Staphylococcus aureus*	2	4.7	0	0.0
Symptomatic UTI	*Yes*	13	28.2	64	55.6
	*No*	33	71.8	51	44.4
Antibiotic resistance	*Yes*	11	23.9	33	28.6
	*No*	35	76.1	82	71.4
ESBL positive	*Yes*	9	19.5	28	25.9
	*No*	37	80.5	80	74.1

RAMACIC: based on the Roy Adaptation Model with an android phone application for patients on clean intermittent catheterization, *CIC* clean intermittent catheterization, *UTI* urinary tract infection, *ESBL* extended spectrum beta-lactamase

## Discussion

Clean intermittent catheterization (CIC) has an integral role in the long-term care of patients with voiding dysfunction who are unable to empty their bladder independently [1, 21]. Caregivers who apply CIC must have sufficient knowledge and skills to be able to completely empty the urine accumulated in the bladder of children using the correct procedure steps. Health professionals who provide training to caregivers in the CIC application process are nurses [7–10, 13, 14]. If caregivers do not have sufficient knowledge/skills regarding CIC application, they cannot perform the application correctly. This increases the risk of developing ITU, which is the most common complication in people who undergo CIC [7–10, 13, 14, 22, 23]. UTIs, if recurrent, can lead to increased hospitalizations, permanent organ damage, and high mortality rates [7, 8, 24, 25]. In order to prevent this situation, many studies, both qualitative and quantitative, have been conducted in the literature on the caregivers of children who underwent CIC. In quantitative studies, it has been clearly emphasized that CIC prevents patients from using permanent bladder catheters in the long term and reduces the frequency of UTI development because it protects bladder and kidney functions [4, 5, 26–28]. It has been emphasized, especially in qualitative studies, that caregivers have difficulty in the CIC learning process, that they are very afraid of making mistakes and harming their children because they do not have sufficient knowledge about the application, and therefore the training given to them is very important in preventing the development of complications, especially UTI, by managing the process positively [29, 30]. In addition, some studies have determined that although caregivers have received CIC training, their skill status regarding application should be monitored at certain intervals [31], and that the incidence of ITU development increases when they cannot place the CIC correctly due to insufficient training [32]. For this reason, it has been particularly emphasized that training for caregivers should be given by nurses who are competent in this regard in accordance with certain protocols/guidelines. However, studies evaluating the effectiveness of the training given to caregivers who implement CIC on caregivers and patients are quite limited in the literature [14]. In a previously conducted randomized controlled experimental study by researchers, it was determined that model-based android application-supported CIC training had a positive contribution to the knowledge/skills of caregivers and to preventing the development of UTI in children [14]. To our knowledge, this study is the first to evaluate the results of model-based and android phone application-supported training over a long period of time, and the data obtained are very valuable. According to the results obtained, it was determined that model-based android phone application-supported (RAMACIC) education for caregivers reduced the development of infection in children in the long term. For this reason, it is very important that the trainings for caregivers suitable for CIC for children are provided by expert nurses in accordance with certain protocols and guidelines; that the trainings are based on nursing theories and models; and that caregivers are supported with android phone applications (RAMACIC) in terms of the frequency of CIC, the correct application of the procedure steps, and attending regular hospital appointments. Thus, as seen in the results obtained from the study, the development of UTI in children can be prevented in the long term, kidney and bladder functions can be protected, and the need for renal replacement therapy, especially hemodialysis, can be reduced.

The literature review also identified a wide range (12–88%) in the incidence of UTI in children with CIC [19, 20]. The use of prophylactic doses of antibiotics to prevent the development of UTI in children undergoing CIC is a controversial issue [3, 7, 25–28, 33]. In the study conducted by Wallis et al. in 2021 with infants diagnosed with SB who underwent CIC, it was determined that the use of prophylactic doses of antibiotics was not necessary to reduce the risk of UTI development in children [25]. Similarly, in a study conducted by Zeger et al. in 2017 with patients diagnosed with SB and undergoing CIC, it was stated that the use of prophylactic doses of antibiotics increased the frequency of resistant bacterial growth in urine and therefore should not be used [33]. On the other hand, in a study conducted by Ben et al. on children who underwent CIC with a diagnosis of neurogenic bladder, it was determined that prophylactic doses of antibiotics could be used based on urine culture test results, and in a study conducted by Jiang et al., it was determined that continuous prophylaxis treatment given to patients who underwent CIC reduced the risk of UTI development [34, 35]. In the study conducted, unlike the literature, the frequency of UTI development, antibiotic resistance, and ESBL positivity were significantly lower in the RAMACIC group despite the regular use of prophylactic doses of antibiotics according to the urine culture test results in both groups. To our knowledge, this is also the first study to investigate the long-term effects of education given to patients undergoing CIC using prophylactic doses of antibiotics on the frequency of UTI development in children. For this reason, the data obtained are very valuable. The results obtained show that the training given by health personnel to caregivers who apply CIC to their children is very important, that the training content should be based on the theories of nursing theorists, and that the care application should be supported by android phone applications.

At this point, the findings obtained regarding the prevention of the risk of UTI development in children with RAMACIC application are consistent with the literature. Studies have emphasized the education of caregivers in order to prevent the development of UTI in children using CIC, and it has been determined that inadequate caregiver education increases the risk of UTI development in patients using CIC. In addition, it has been determined that the frequency of UTI development decreases when caregivers are adapted to the process they live by nurses, use non-traumatic techniques during CIC application, apply CIC using the correct procedure steps, and pay attention to the average daily catheterization number and frequency [1, 4, 11]. RAMACIC, which is implemented by researchers, enables the caregivers to adapt to the process they are experiencing, as well as the education of caregivers, and at the same time, thanks to the android phone application, it enables caregivers to apply CIC at the frequency specified by the doctor, using the correct process steps according to gender.

In this context, the educational model referred to as RAMACIC has two basic components. One of these components is the training of caregivers in line with the RAM created by Sister Callista Roy, a nurse theorist, and the other is the application of CIC at the frequency determined by the doctor, using the correct process steps with the developed android phone application. For this reason, in regions where smartphone access is limited within the scope of RAMACIC, while the training for caregivers is given by nurses, illustrated training booklets and brochures should be prepared and delivered to the caregivers at the end of the training so that the caregivers can use the correct procedure steps.

The study also evaluated the type of bacteria that caused UTI in both groups. According to the results, it was determined that the most frequently growing bacteria in both groups was *E. coli*. In addition, it was determined that this growth was at a lower rate in the RAMACIC group. The study underscores the importance of caregiver knowledge and skills in hygiene practices, particularly in preventing the proliferation of *E. coli*, a gram-negative bacterium known to elevate infection risks in children due to its excretion-preventing fimbriae. The findings are consistent with the literature. Many studies in the literature have determined that the most common bacterial species in patients receiving CIC is *E. coli*. Studies have determined that the Gram-negative bacteria *E. coli* increases the risk of developing UTI in children due to its fimbriae that prevent its excretion [36–39]. Therefore, the importance of cleaning the genital area using the correct procedure steps appropriate for the child’s gender, especially hand washing, should be emphasized in the content of the training given to caregivers.

## Conclusion

This study is the first to evaluate the long-term effect of model-based android phone application-supported education on the development of UTI in children with CIC. The primary result of the study is that CIC education facilitated through an android phone application compatible with theoretical frameworks is effective in reducing the frequency of UTI in patients in the long term. In addition, RAMACIC was found to be effective in preventing antibiotic resistance and ESBL positivity in patients. In line with the results obtained from the study, it is thought that RAMACIC will reduce the frequency of children developing UTI, thus contributing to the reduction of the frequency of return to health institutions and the costs within the scope of health services. In addition, RAMACIC is thought to contribute positively to the quality of life of both children and their caregivers as a result of the reduction in the frequency of renal replacement therapy due to permanent organ damage following UTI, the most common complication in children treated with CIC. As a result, it is important to improve caregiver education using android phone applications that are compatible with nursing theories and broader theoretical frameworks, especially to improve knowledge and skills related to invasive interventions that require practical competence.

## Limitations of the study

Limitations of the study include its lack of generalizability, as the findings are specific to caregivers of patients undergoing CIC in the pediatric nephrology outpatient clinic of a university hospital in a particular region. Additionally, the scarcity of relevant literature constrains a thorough discussion of the study results.

## Data Availability

No datasets were generated or analysed during the current study.
